# IR/IGF1R signaling as potential target for treatment of high-grade osteosarcoma

**DOI:** 10.1186/1471-2407-13-245

**Published:** 2013-05-20

**Authors:** Marieke L Kuijjer, Elisabeth FP Peterse, Brendy EWM van den Akker, Inge H Briaire-de Bruijn, Massimo Serra, Leonardo A Meza-Zepeda, Ola Myklebost, A Bassim Hassan, Pancras CW Hogendoorn, Anne-Marie Cleton-Jansen

**Affiliations:** 1 Department of Pathology, Leiden University Medical Center, Albinusdreef 2, Leiden 2300RC, the Netherlands; 2 Laboratory of Experimental Oncology Research, Istituto Ortopedico Rizzoli, Via G.C. Pupilli 1, Bologna 40136, Italy; 3 Department of Tumor Biology, the Norwegian Radium Hospital, Oslo University Hospital, Montebello, Oslo 0310, Norway; 4 Sir William Dunn School of Pathology, University of Oxford, South Parks Road, Oxford OX1 3RE, UK

**Keywords:** Osteosarcoma, IGF1R signaling, Signal transduction, IGF1R, OSI-906, Bone neoplasm, Sarcoma

## Abstract

**Background:**

High-grade osteosarcoma is an aggressive tumor most often developing in the long bones of adolescents, with a second peak in the 5th decade of life. Better knowledge on cellular signaling in this tumor may identify new possibilities for targeted treatment.

**Methods:**

We performed gene set analysis on previously published genome-wide gene expression data of osteosarcoma cell lines (n=19) and pretreatment biopsies (n=84). We characterized overexpression of the insulin-like growth factor receptor (IGF1R) signaling pathways in human osteosarcoma as compared with osteoblasts and with the hypothesized progenitor cells of osteosarcoma – mesenchymal stem cells. This pathway plays a key role in the growth and development of bone. Since most profound differences in mRNA expression were found at and upstream of the receptor of this pathway, we set out to inhibit IR/IGF1R using OSI-906, a dual inhibitor for IR/IGF1R, on four osteosarcoma cell lines. Inhibitory effects of this drug were measured by Western blotting and cell proliferation assays.

**Results:**

OSI-906 had a strong inhibitory effect on proliferation of 3 of 4 osteosarcoma cell lines, with IC_50_s below 100 nM at 72 hrs of treatment. Phosphorylation of IRS-1, a direct downstream target of IGF1R signaling, was inhibited in the responsive osteosarcoma cell lines.

**Conclusions:**

This study provides an *in vitro* rationale for using IR/IGF1R inhibitors in preclinical studies of osteosarcoma.

## Background

High-grade osteosarcoma is the most prevalent primary malignant bone tumor. The disease occurs most frequently in children and adolescents at the site where proliferation is most active, *ie* the metaphysis adjacent to the epiphyseal plate [1]. The 5-year overall survival of osteosarcoma patients has raised from 10-20% to about 60% after the introduction of preoperative chemotherapy in the 1970s. However, about 45% of all patients still die because of distant metastasis. No additional treatments have been found that can increase survival significantly, and administering higher doses of preoperative chemotherapy does not result in improved outcomes [2,3]. Better knowledge on cellular signaling in high-grade osteosarcoma may identify new possibilities for targeted treatment of this highly aggressive tumor.

We have previously described the roles of bone developmental pathways Wnt, TGFβ/BMP, and Hedgehog signaling in osteosarcoma, but unfortunately so far could not identify suitable targets for treatment [4,5]. In addition to these signal transduction pathways, insulin-like growth factor 1 receptor (IGF1R) signaling plays a key role in the growth and development of bone. Aberrant signaling of this pathway has been implicated in various cancer types, among others sarcomas [6,7]. Key players of insulin-like growth factor (IGF) signaling are the ligands IGF1, IGF2, which are circulating polypeptides that can be expressed in endocrine, paracrine, and autocrine manners, and the tyrosine kinase receptor IGF1R, which forms homodimers, or hybrid receptors with the insulin receptor (IR) [8]. IGF1R and IR/IGF1R hybrids are activated by both IGF1 and −2, which trigger autophosphorylation of IGF1R and subsequent downstream signal transduction. A second IGF receptor, IGF2R, can bind IGF2, but does not confer intracellular signaling, thereby diminishing the bioavailability of IGF2 to IGF1R [9]. Autophosphorylation of IR/IGF1R receptors recruits the signaling proteins insulin receptor substrate (IRS) and Src homology 2 domain containing transforming protein (Shc) to the cell membrane, which get phosphorylated and subsequently activate the downstream PI3K/Akt and Ras/Raf/ERK signaling pathways, both of which are known to be important in cancer. These pathways ultimately act on several biological processes, such as transcription, proliferation, growth, and survival [9-11]. Interestingly, treatment targeted against IGF1R signaling has shown to be effective in a subset of Ewing sarcoma, another bone tumor that manifests at young age [12].

The role of the IGF1R pathway in growth has been illustrated in studies of knockout mice. It was shown that IGF1 null mice are 40% smaller than littermates, while IGF1R null mice are approximately 55% smaller [13]. In dogs, the size of different breeds was demonstrated to be dependent on IGF1 plasma levels [7]. Additionally, a specific IGF1 SNP haplotype was described to be common in small breed dogs and nearly absent in giant breeds [14]. Interestingly, large and giant dog breeds are more prone to develop osteosarcoma [15], which in dogs is biologically very similar to the human disease [16]. Two recent studies on human osteosarcoma suggest a positive correlation between patient birth-weight and height at diagnosis and the development of the disease [17,18]. Involvement of some members of IGF1R signaling in osteosarcoma has been described (as has been reviewed in Kolb *et al*. [19]), but the activity of this pathway remains to be determined.

We have analyzed genome-wide gene expression in high-grade osteosarcoma cell lines and pretreatment biopsies, and observed significantly altered activity of genes involved in IGF1R signaling when compared to profiles of mesenchymal stem cells and osteoblasts. Specifically, upstream inhibitors of IGF1R signaling were found to be downregulated in osteosarcoma, and low expression of these genes correlated with worse event-free survival. We inhibited IR/IGF1R signaling with the dual IR/IGF1R inhibitor OSI-906. This showed inhibition of phosphorylation of IRS-1 and of strong inhibition of proliferation in 3/4 osteosarcoma cell lines. Interestingly, the cell line which could not be inhibited with OSI-906, 143B, has a k-ras oncogenic transformation, which is a component of the Ras/Raf/ERK pathway, one of downstream effectors of IGF1R signaling. These results suggest that IR/IGF1R signaling may be an effective targeted for treatment of high-grade osteosarcoma patients.

## Methods

### Cell culture

The 19 high-grade osteosarcoma cell lines that were used in this study were characterized and are described in Ottaviano *et al*. [20]. The 12 mesenchymal stem cell and 3 osteoblast cultures were previously described [21]. MSCs have been previously [22] characterized through FACS analysis and have been tested for their ability to be committed under proper conditions towards adipogenesis, chondrogenesis and osteogenesis as described in Bernardo *et al*. [23]. Osteoblast cultures were derived from MSCs which were treated to undergo osteogenic differentiation. Cell line DNA was short tandem repeat profiled to confirm cell line identity with use of the Cell ID system of Promega (Madison, WI). For Western blotting experiments, cells were maintained in RPMI 1640 (Invitrogen, Carlsbad, CA), supplemented with 10% fetal bovine serum (F7524, Sigma-Aldrich, St. Louis, MO) and 1% glutamax (Gibco 35050, Invitrogen, Carlsbad, CA).

### Microarray experiments, preprocessing, and data analysis

For genome-wide gene expression analysis, we used Illumina Human-6 v2.0 BeadChips. Microarray experiments and data preprocessing are described in Kuijjer *et al*. [21]. Previously deposited genome-wide gene expression data of mesenchymal stem cells (MSCs) and osteoblasts can be found in the Gene Expression Ombinus (GEO accession number GSE28974 and GSE33382, respectively). Data from osteosarcoma cell lines have been published before [24], but since we normalized and processed all raw data together, we deposited normalized values in the Gene Expression Omnibus (GEO, accession number GSE42351, superseries accession GSE42352). Data from the 84 high-grade osteosarcoma pretreatment biopsies have been previously published (GEO accession number GSE33382) [21]. Ethical guidelines of the individual European partner institutions were followed and samples and clinical data were handled in a coded fashion and stored in the EuroBoNeT biobank. We determined significant differential expression between osteosarcoma cell lines (n=19) and mesenchymal stem cells (n=12), and between osteosarcoma cell lines and osteoblasts (n=3) using Bioconductor [25] package *LIMMA*[26] in statistical language R [27]. Probes with Benjamini and Hochberg false discovery rate-adjusted *P*-values <0.05 were considered to be significant. Gene set analysis was performed on KEGG pathways [28] (Release 63.0, July 1, 2012) using R-package globaltest [29]. For each analysis, the top 15 significant KEGG pathways were returned. All returned pathways had a Benjamini and Hochberg false-discovery rate-corrected *P*-value <1·10^-5^. To visualize differential expression in the IGF1R pathway, we performed Core analyses using Ingenuity Pathways Analysis (IPA, Ingenuity Systems, http://www.ingenuity.com).

### Antibodies and reagents

Rabbit monoclonal and polyclonal antibodies against IGF1R and IRS-1, respectively (both 1:1,000) were obtained from Cell Signaling (Danvers, MA). Rabbit polyclonal antibody against phospho-IRS-1 (Y612, 1:1,000) was purchased from Biosource, Invitrogen (Carlsbad, CA). A mouse monoclonal antibody against α-tubulin from Abcam (Cambridge, UK) was used as a loading control (1:3,000). Secondary antibodies (both 1:10,000, BD Transduction Laboratories, Lexington, KY) were horseradish peroxidase (HRP) conjugated polyclonal goat-anti-rabbit IgG for components of the IR/IGF1R pathway, and HRP conjugated polyclonal goat-anti-mouse for α-tubulin. OSI-906 was purchased from Selleck Chemicals LLC (Houston, TX).

### Western blotting

Osteosarcoma cell lines OHS, KPD, SAOS2, and 143B were treated with 0.5% DMSO or with 1 μM OSI-906 for 3 hrs, and were subsequently lysed using Mammalian Protein Extraction Reagent (Thermo Scientific 78503), to which Halt Phosphatase and Protease Inhibitor Cocktails (Thermo Scientific 78420 and 78418, respectively) were added according to the manufacturer’s protocol. Concentrations of cell lysates were determined using the BioRad DC Protein Assay Kit (Biorad, Hercules, CA). Per sample, 20 μg of protein was loaded on SDS-PAGE gels. Lysate of HepG2-A16 cells transfected with IR and stimulated with insulin, containing 10 μg of protein, was taken along as a positive control. Western blotting was performed as described in Schrage *et al*. [30].

### Proliferation assays

OSI-906 was diluted in DMSO and stored at −20°C. OHS, SAOS2, KPD, and 143B cells were plated in 96 wells plates, using 4,000, 2,000, 12,000, and 2,000 cells per well, respectively. After 24 hrs, OSI-906 was added in triplicate at different concentrations – 0 nM, 0.01 nM, 0.1 nM, 1 nM, 10 nM, 100 nM, 1 μM, and 10 μM. The inhibitor was incubated for 72 hrs and 96 hrs, in different experiments. The results shown are representative results from at least three independent experiments. Cell proliferation reagent WST-1 (Roche) was incubated for 2 hrs and subsequently measured using a Wallac 1420 VICTOR2 (Perkin Elmer, Waltham, MA). Data were analyzed in Graphpad Prism 5.0 (http://www.graphpad.com). Relative IC_50_s were calculated using results from the different concentrations up to the highest dose where toxicity was not yet present.

## Results

### Enrichment of IGF1R signaling in high-grade osteosarcoma

Genome-wide gene expression data were of good quality for all cell lines. *LIMMA* analysis resulted in 7,891 probes encoding for differentially expressed (DE) genes between osteosarcoma cell lines and MSCs, and 2,222 probes encoding for DE genes between osteosarcoma cells and osteoblasts. We tested the global expression patterns of KEGG pathways using globaltest [29] and determined the intersection of the pathways most significantly different in osteosarcoma cell lines as compared with MSCs, and of osteosarcoma cell lines as compared with osteoblasts. This approach resulted in five significantly affected pathways – insulin signaling pathway, oocyte meiosis, ubiquitin mediated proteolysis, progesterone-mediated oocyte maturation, and glycerophospholipid metabolism. Details of the globaltest are shown in Table 1. IGF1R signaling is involved in three out of the five detected KEGG pathways (insulin signaling pathway, oocyte meiosis, and progesterone-mediated oocyte maturation). Interestingly, a globaltest on mRNA expression of previously published pretreatment biopsies [21] compared with normal bones [31]) also returned insulin signaling as the most significantly affected pathway (*data not shown*). Notably, there is no specific IGF1R signaling pathway in the KEGG database [28]. Because of the over-representation of IGF1R signaling, and because of its known role in cancer, we decided to study expression of members of this pathway in detail.

**Table 1:** Globaltest results

**KEGG pathway**	**Analysis**	**adj*P***	**Statistic**	**Expected**	**Std**.**dev**
Insulin signaling pathway	OScellvsOB	1.01∙10^-7^	26.34	4.76	1.92
	OScellvsMSC	3.07∙10^-15^	35.12	3.33	1.78
Oocyte meiosis	OScellvsOB	2.70∙10^-7^	37.45	4.76	2.9
	OScellvsMSC	5.04∙10^-16^	53.7	3.33	2.84
Ubiquitin mediated proteolysis	OScellvsOB	3.21∙10^-7^	22.88	4.76	1.75
	OScellvsMSC	5.04∙10^-16^	37.99	3.33	1.89
Progesterone-mediated oocyte maturation	OScellvsOB	7.16∙10^-7^	34.26	4.76	2.71
	OScellvsMSC	1.34∙10^-15^	55.35	3.33	2.77
Glycerophospholipid metabolism	OScellvsOB	1.40∙10^-6^	27.13	4.76	2.25
	OScellvsMSC	2.25∙10^-15^	55.86	3.33	2.82

The top five significant pathways with aberrant expression in both osteosarcoma cell lines versus osteoblasts (OScellvsOB) and osteosarcoma cell lines versus mesenchymal stem cells (OScellvsMSC). adjP: FDR-adjusted p-value, Statistic: test statistic of the globaltest, Expected: expected test statistic of the globaltest, Std.dev: standard deviation under the null hypothesis.

### Differentially expressed genes of the IGF1R pathway

**Figure 1:**
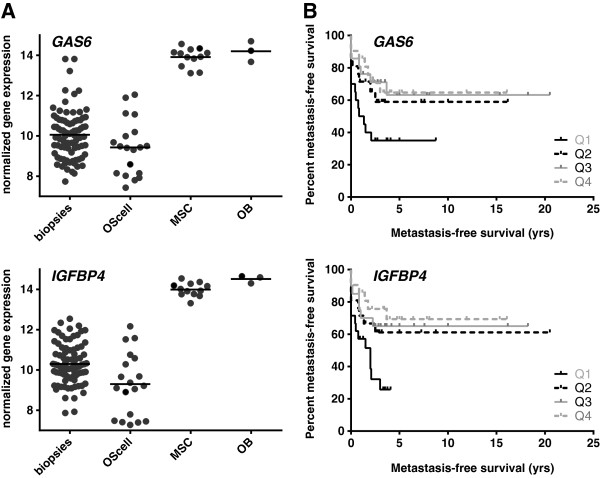
**mRNA expression of *GAS6 *and *IGFBP4*. *A ***Normalized gene expression levels of *GAS6* and *IGFBP4* in osteosarcoma biopsies, cell lines, mesenchymal stem cells (MSCs), and osteoblasts (OB). Expression of both proteins is considerably higher in the controls (FDR-adjusted *P*<0.001 for both genes in all four analyses). ***B ***Kaplan-Meier curves depicting metastasis-free survival in years for 83 high-grade osteosarcoma patients (for 1/84 patients, we did not have follow-up data available), based on quartiles of mRNA expression of the genes of interest.

**Figure 2:**
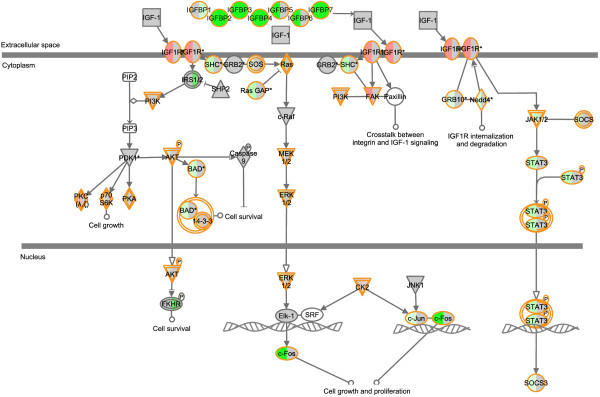
**Ingenuity pathways analysis canonical pathway IGF1 signaling** This figure shows the IGF1 signaling pathway, with significantly upregulated genes in red, downregulated genes in green, and genes that did not meet our criteria for significance in gray. The left part of the symbols shows the analysis of osteosarcoma cell lines as compared with mesenchymal stem cells, the right part as compared with osteoblasts. Most consensus in gene expression is found upstream IGF1R signaling, in the expression of the IGF binding proteins.

To determine which genes have the most specific up- or downregulation in osteosarcoma, we combined lists of significantly differentially expressed genes of osteosarcoma cell lines (n=19) and a previously published set of osteosarcoma pretreatment biopsies (n=84, GEO accession GSE33382) in comparison with mesenchymal stem cells (n=12) and osteoblasts (n=3) by four-way Venn analysis of all significantly affected probes with the same direction of fold change (upregulated or downregulated in all four analyses) (Additional files 1 and 2). We identified *IGFBP4* and *GAS6* as the most downregulated genes in osteosarcoma (average log fold changes of -4.43 and -4.29, respectively). *IGFBP2* was also present in the top 20 results from this four-way analysis (see Additional file 1). In addition, *IGFBP3* and −*7* were significantly downregulated, and *IGF2BP3* was significantly upregulated in three out of the four analyses. Both *IGFBP4* and *GAS6* show high variability in expression in osteosarcoma cell lines and biopsies (Figure 1*A*). Patients of whom biopsies had very low expression of these genes had poor event-free survival profiles (log-rank test for trend, *P* = 0.01 for *IGFBP4* and *P* = 0.04 for *GAS6*, Figure 1*B*). To visualize mRNA expression of the IGF1R signaling pathway members, we used Ingenuity Pathways Analysis on *LIMMA* toptables from osteosarcoma cells as compared with mesenchymal stem cells and from osteosarcoma cells as compared with osteoblasts (Figure 2). As can be seen in this figure, overlap of differentially expressed genes between these analyses was detected upstream of IGF1R.

### OSI-906 inhibits phosphorylation of IRS-1

**Figure 3:**
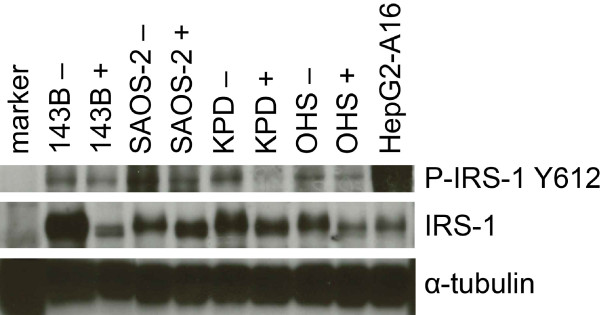
**Validation of IR**/**IGF1R downstream signaling.** Western blot of IRS-1 and p-IRS-1 of lysates of untreated (−) osteosarcoma cell lines OHS, KPD, SAOS2, and 143B, and of these cells treated for 3 hrs with 1 μM of OSI-906 (+).

Gene expression levels of IGF1R and IRS-1 were validated at the protein level by Western blot analysis (Additional file 3). To determine the activity of IR/IGF1R signaling, we performed Western blot analysis on cell lysates of OHS, KPD, SAOS2, and 143B, using antibodies against IRS-1 and phosphorylated IRS-1, before and after treatment with OSI-906, a selective small molecule dual kinase inhibitor of both IR and IGF1R, as IRS-1 is a direct downstream target of IGF1R. An inhibition of intrinsic IRS-1 phosphorylation at Y612 was detected after treatment with OSI-906 in all cell lines (Figure 3), indicating that this inhibitor could affect signaling downstream IGF1R in osteosarcoma cells.

### OSI-906 inhibits proliferation of 3 of 4 osteosarcoma cell lines

**Figure 4:**
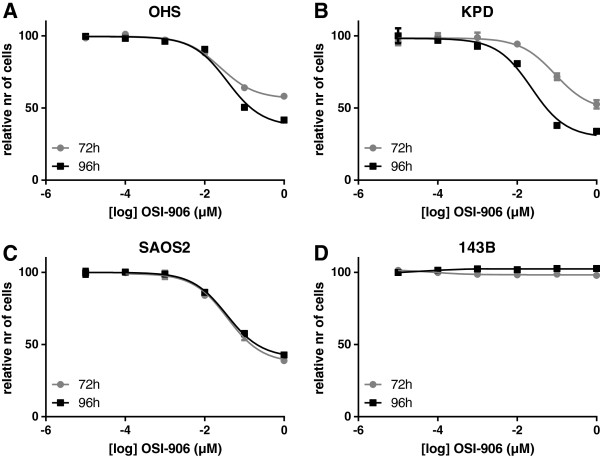
**Inhibition of osteosarcoma cell lines with OSI**-**906.** Osteosarcoma cell lines were inhibited with different concentrations of OSI-906, for 72 (gray line) or 96 (black line) hours. OHS (***A***), KPD (***B***), and SAOS2 (***C***) showed a dose-dependent inhibition, while 143B (***D***) did not respond to OSI-906.

In 3 of 4 osteosarcoma cell lines tested, inhibition with OSI-906 was dose-dependent (Figure 4). Except for a toxic response at the maximum dose of 10 μM (Additional file 4), there was no effect on 143B. Because of this toxicity, relative IC_50_s were determined using measurements until 1 μM. OHS, SAOS2, and KPD had an IC_50_ of 25 nM, 92 nM, and 90 nM at 72h, respectively, and of 37 nM, 57 nM, and 23 nM at 96h of inhibition, respectively. At 1 μM OSI-906, approximately 60% of proliferation of OHS, SAOS2, and KPD cells was inhibited, while 143B proliferation was not inhibited (Figure 4).

## Discussion

Genome-wide gene expression and subsequent gene set analysis on osteosarcoma cell lines and biopsies indicated increased insulin-like growth factor signaling in high-grade osteosarcoma as compared with the hypothesized osteosarcoma progenitors, which is currently the best control, since there is no benign precursor and no certainty of the normal counterpart of osteosarcoma. Because IGF1R signaling can be exploited as a therapeutic target, and osteosarcoma patients are in severe need of new therapies, we examined mRNA expression of members of this signaling pathway in detail. *IGFBP4* and *GAS6*, which code for proteins that inhibit IGF1R signaling, showed the highest significant downregulation (log fold changes <−4) in a four-way analysis, in which osteosarcoma pretreatment biopsies or cell lines were compared with osteoblastic cultures (n=3) or MSCs (n=12). Insulin-like growth factor binding proteins (IGFBPs) generally inhibit IGF1R signaling by competitively binding IGFs, but can under certain circumstances also stimulate IGF1R signaling [32]. IGFBP4 is a negative regulator of IGF signaling in various tissues, including bone [33]. GAS6, or growth arrest-specific 6, was shown to inhibit the growth promoting effects of IGF signaling and to stimulate differentiation in the chondrogenic cell line ATDC5 [34]. Both *IGFBP4* and *GAS6* expression have previously been shown to be downregulated in osteosarcoma cell lines (*IGFBP4* in MG63 [35], *GAS6* in MG63 and SAOS2 cells [36]). Next to *GAS6* and *IGFBP4*, *IGFBP2* was also significantly downregulated in all four analyses, with log fold changes of approximately -3. IGFBP2 generally inhibits IGF action and may play a role in IGF2-induced osteoblast differentiation [33]. *IGFBP3* was highly downregulated in three out of four analyses, and has been shown to elicit anticancer effects by inhibiting IGF1R signaling in Ewing sarcoma [37]. IGFBP7 activity has not yet been reported in sarcoma, but has been associated with *e*.*g*. hepatocellular carcinoma [38]. Interestingly, *IGF2BP3* was highly overexpressed in 3 of 4 analyses. This binding protein can bind IGF2 mRNA, thereby probably activating the translation of IGF2 [39]. Overexpression of *IGF2BP3* has been reported in several cancer types [40,41]. Figure 2 shows that differential expression is most pronounced in upstream regulators of IGF1R, while downstream components, such as *SHC* and *FOS*, are slightly downregulated, although for most genes this only holds when compared with mesenchymal stem cells, and not with osteoblasts. This may be caused by negative feedback loops, triggered by the active IGF1R signaling pathway. These results suggest that, in osteosarcoma, the IGF1R signaling pathway can be inhibited at the level of the receptor. We therefore validated protein levels of IGF1R and of IRS-1, a direct downstream component of IGF1R and IR signaling using Western blotting. IGF1R and IRS-1 protein levels correlated fairly well with mRNA expression levels. Most importantly, phosphorylated IRS-1, which is a measure for pathway activity, was detected in all four osteosarcoma cell lines, indicating that IGF1R signaling is active in osteosarcoma, and is possibly regulated upstream of IGF1R. Accordingly, targeting this receptor may be an effective way to inhibit this pathway.

OSI-906 is a selective small molecule dual kinase inhibitor of both IR and IGF1R [42]. We specifically chose to treat osteosarcoma cells with a dual inhibitor, because the insulin receptor can activate the same downstream signaling pathways as IGF1R, therefore providing cells a way to circumvent single inhibition of IGF1R. This has formerly been demonstrated in osteoblasts [43] and in Ewing sarcoma cells [44]. In fact, this dual inhibitor has been shown to cause enhanced inhibition of the Akt signaling pathway when compared with a selective monoclonal antibody against IGF1R, which could inhibit IR/IGF1R hybrids, but not IR homodimers [45]. OSI-906 is currently being tested by OSI Pharmaceuticals in a Phase III trial in adrenocortical carcinoma and in a Phase I/II clinical trial in ovarian cancer. Treatment of osteosarcoma cells with OSI-906 at physiological levels leads to decreased phosphorylation of IRS-1 at Y612. Inhibition of IRS-1 at Y612 after treatment with OSI-906 was previously reported by Buck *et al*. in direct complementation breast cancer cells for IGF1R-IGF2 and IR(A)-IGF2 [45]. Interestingly, we also detected a small shift in the size of p-IRS-1 on the Western Blot, indicating that multiple phosphorylation groups are removed after treatment with OSI-906. Surprisingly, total IRS-1 levels were highest in 143B, and were downregulated after treatment with OSI-906 in this cell line, although this had no effect on cell growth in this line, as opposed to the three others, which showed low IC_50_s. Proliferation of 143B was only inhibited most likely unspecifically at high and toxic levels of the drug. The 143B cell line is a derivative of the osteosarcoma cell line HOS, transformed by a *KRAS* oncogene. Constitutive activation of the Ras/Raf/ERK pathway can explain why proliferation of this cell line cannot be inhibited by OSI-906. Of the cell lines that were responsive to OSI-906, KPD and OHS showed that treatment of 96 hrs was most effective, while SAOS2 already reached maximum inhibition at 72 hrs.

IGF1R signaling has been previously modulated in sarcoma in preclinical and clinical models. Several phase I and II clinical trials including treatment with IGF1R monoclonal antibodies are currently being conducted in sarcoma, especially in Ewing sarcoma (an overview of these trials is given in Olmos *et al*. [46]). Monoclonal antibodies against IGF1R have modest activity against Ewing sarcoma, as was observed in a phase I/II study of figitumumab (partial response in 14.2% of all subjects) [47] and in a phase II study using R1507 (complete/partial response rate of 10%) [48]. Results of a phase II study of ganitumab in subjects with Ewing sarcoma and desmoplastic small round cell tumors were published very recently, and reported clinical benefit in 17% of all patients [49]. Preclinically, treatment with different monoclonal antibodies against IGFR1 has been performed in osteosarcoma xenograft models, in which a response was detected in at least 60% of all cases studied [50-52]. However, no objective responses were observed in phase I trials testing monoclonal antibodies [47,53,54], although 2 of 3 patients treated with R1507 had prolonged stable disease [53]. Clinical data using dual IGF1R/IR inhibitors osteosarcoma is still very limited [55]. Because resistance to highly specific IGF1R inhibitors may develop through IR [44], blocking both IGF1R and IR with a dual kinase inhibitor will most likely lead to better inhibition of downstream IRS-1 signaling. We thus expect clinical outcomes to improve for osteosarcoma patients treated with dual IGF1R/IR inhibitor OSI-906. The effects of combination of OSI-906 with chemotherapeutics in osteosarcoma still need to be assessed before such a treatment can be clinically tested.

Phosphorylated IRS could be used as a biomarker in order to determine whether patients would respond to IGF1R inhibition. Patients with tumors exhibiting an activating mutation in downstream pathways will most likely not respond to IGF1R inhibition. Further research needs to be performed in order to assess these candidate biomarkers for response to treatment. The IGF1R pathway acts on several biological mechanisms that promote tumor progression – mitogenesis, protection from apoptosis, malignant transformation, and metastasis [6]. It is therefore possible that inhibiting these pathways with a dual IR/IGF1R kinase inhibitor, such as OSI-906, may reduce tumor sizes, as well as osteosarcoma metastasis, the leading cause of death in these patients.

## Conclusions

Using gene set analysis of genome-wide gene expression data of high-grade osteosarcoma biopsies and cell lines, we detected an over-representation of IGF1R signaling. Specifically, different upstream inhibitors of IGF1R signaling, *eg* several IGF binding proteins, were downregulated. As this indicated the IGF1R receptor as a potential target for treatment of osteosarcoma, we set out to inhibit this receptor in four osteosarcoma cell lines. We used OSI-906, a selective small molecule dual kinase inhibitor of both IR and IGF1R, since the insulin receptor can activate the same downstream signaling pathways as IGF1R, thereby providing a way to circumvent single inhibition of IGF1R. Treatment with OSI-906 resulted in inhibition of phosphorylation of IRS-1 Y612, a direct downstream target of IGF1R, and in strong inhibition of proliferation in 3 of 4 osteosarcoma cell lines. The non-responsive cell line, 143B, has a k-ras oncogenic transformation, and may therefore not respond to this treatment. In conclusion, we have shown that IGF1R signaling is active in osteosarcoma, and that dual inhibition of IR/IGF1R inhibits downstream signaling and proliferation of these cells. Responsiveness to this treatment may be evaluated by Western blotting against phosphorylated IRS. This study provides an *in vitro* rationale for using dual IR/IGF1R inhibitors in preclinical studies of osteosarcoma.

## Abbreviations

DE: Differentially expressed; HRP: Horseradish peroxidase; IGF: Insulin-like growth factor; IGF1R: Insulin-like growth factor receptor; IGFBPs: Insulin-like growth factor binding proteins; IPA: Ingenuity Pathways Analysis; IR: Insulin receptor; IRS: Insulin receptor substrate; KEGG: Kyoto encyclopedia of genes and genomes; MSC: Mesenchymal stem cell; OB: Osteoblast; Shc: Src homology 2 domain containing transforming protein.

## Competing interests

The authors declare that they have no competing interests.

## Authors’ contributions

MLK performed all bioinformatics analysis and wrote the manuscript. EFPP, IHB, BEWMA performed Western blotting experiments. EFPP and BH performed inhibition studies. MS, LAMZ, and OM and were involved collection of cell line data. AMC, PCWH, BH, and MLK designed the study. All authors read and approved the final version of the manuscript.

## Pre-publication history

The pre-publication history for this paper can be accessed here:

http://www.biomedcentral.com/1471-2407/13/245/prepub

## Supplementary Material

Additional file 1 Result from the four-way intersection of differentially expressed probes with same direction of fold change (all up- or all downregulated). Click here for file

Additional file 2 **Four-way Venn diagram depicting *A *the number of significantly differentially expressed probes in all four analyses *B *the number of significantly differentially expression probes with same direction of fold change in all four analyses (all up- or all downregulated).** In total, we detected 495 probes that were significant in all analyses. 487/495 significant probes had the same direction of fold change in all four analyses. Cell*vs*OB: osteosarcoma cell lines vs osteoblasts, CellvsMSC: osteosarcoma cell lines vs MSCs, Biop*vs*OB: osteosarcoma biopsies vs osteoblasts, Biop*vs*MSC: osteosarcoma biopsies vs MSCs. Click here for file

Additional file 3 **Validation of expression levels or IGF1R and IRS-1. ***A* Normalized expression levels of IGF1R and IRS-1 in the panel of 19 osteosarcoma cell lines. For both genes, we selected cell lines with relatively low and high mRNA expression (black dots), and determined protein levels on cell lysates using Western blotting. *B* Western blotting results of the selected cell lines. Click here for file

Additional file 4 Dose response curves up to toxic levels of OSI-906. Osteosarcoma cell lines were inhibited with different concentrations of OSI-906, for 72 (gray line) or 96 (black line) hours. Click here for file
