# Supplementary figures and images for: IR/IGF1R signaling as potential target for treatment of high-grade osteosarcoma
Source: BMC Cancer. 2013 May 20;13:245. doi: 10.1186/1471-2407-13-245 (PMC3672007; doi:10.1186/1471-2407-13-245)

## Supplemental Figure 1

**A**

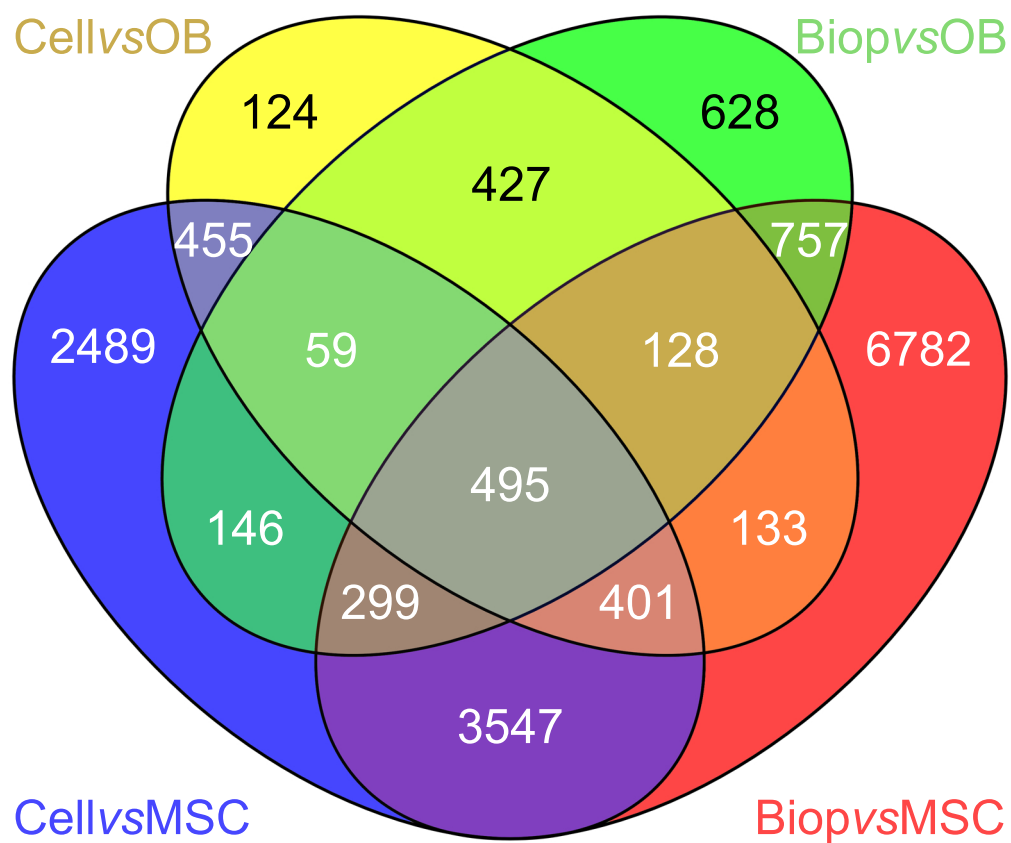

**B**

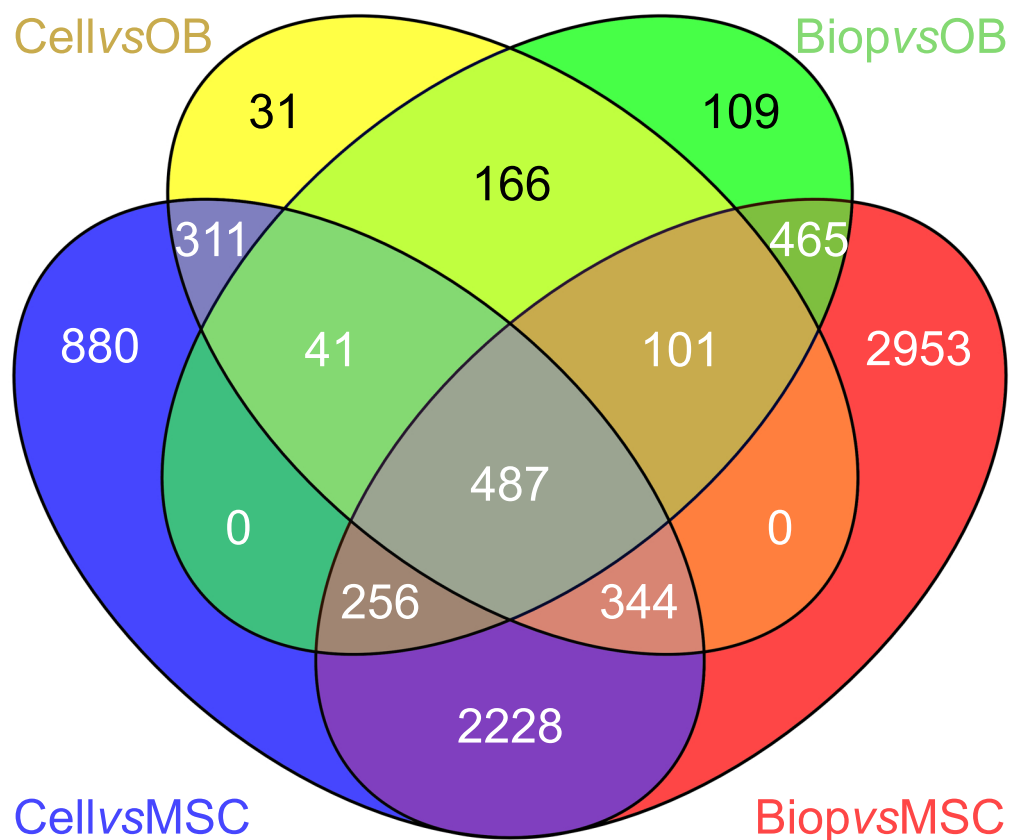

Supplement: Additional file 2 — Four-way Venn diagram depicting A the number of significantly differentially expressed probes in all four analyses B the number of significantly differentially expression probes with same direction of fold change in all four analyses (all up- or all downregulated). In total, we detected 495 probes that were significant in all analyses. 487/495 significant probes had the same direction of fold change in all four analyses. CellvsOB: osteosarcoma cell lines vs osteoblasts, CellvsMSC: osteosarcoma cell lines vs MSCs, BiopvsOB: osteosarcoma biopsies vs osteoblasts, BiopvsMSC: osteosarcoma biopsies vs MSCs. [file 1471-2407-13-245-S2.pdf]

## Supplemental Figure 2

**A**

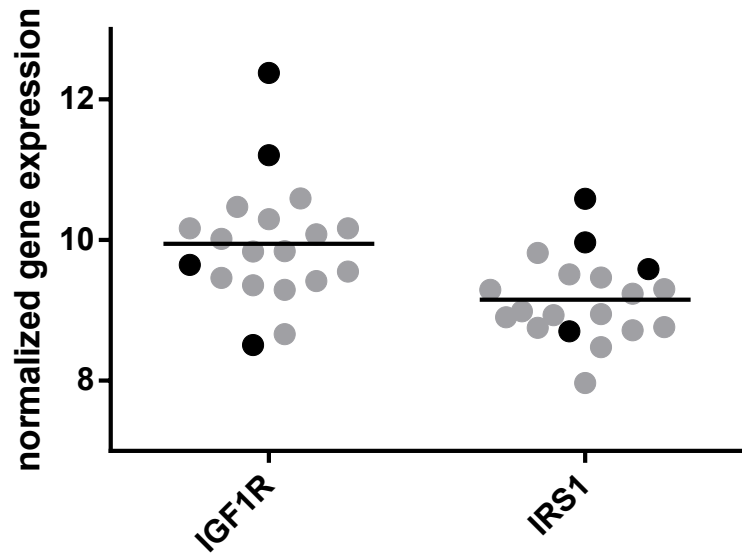

# B

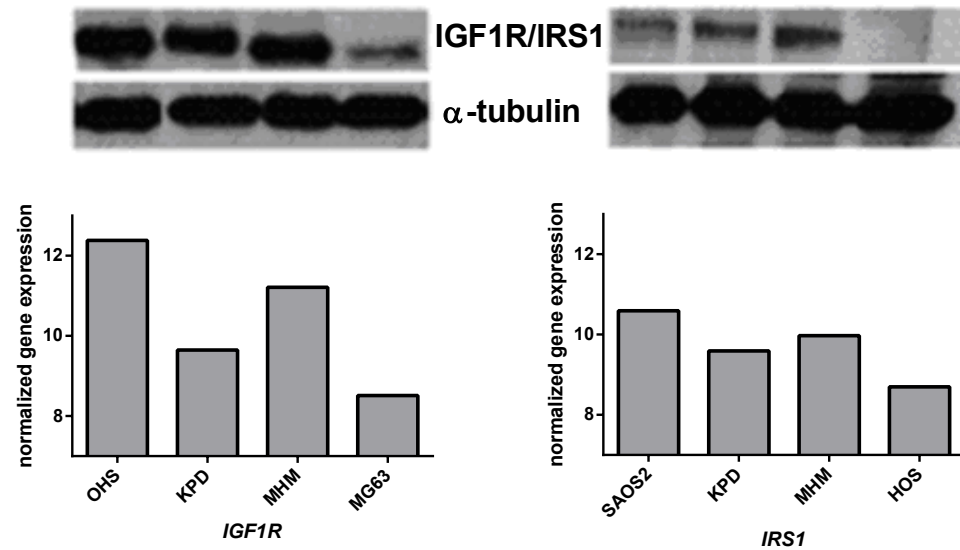

Supplement: Additional file 3 — Validation of expression levels or IGF1R and IRS-1. A Normalized expression levels of IGF1R and IRS-1 in the panel of 19 osteosarcoma cell lines. For both genes, we selected cell lines with relatively low and high mRNA expression (black dots), and determined protein levels on cell lysates using Western blotting. B Western blotting results of the selected cell lines. [file 1471-2407-13-245-S3.pdf]

Supplemental Figure 3

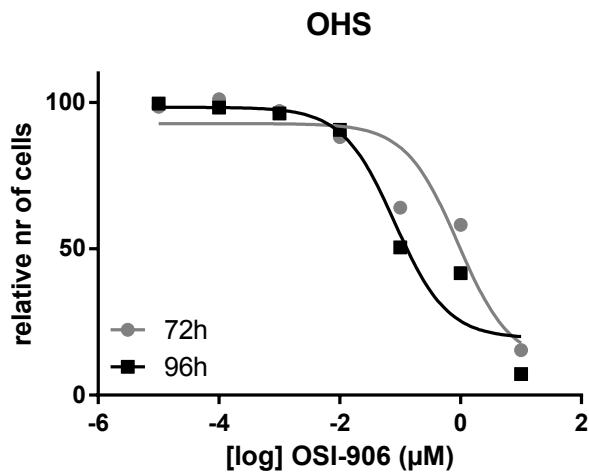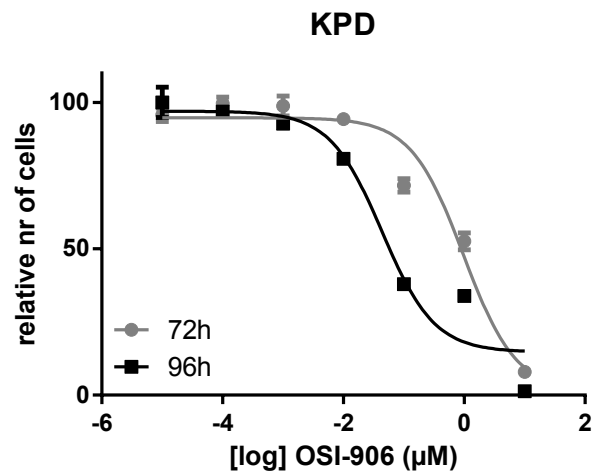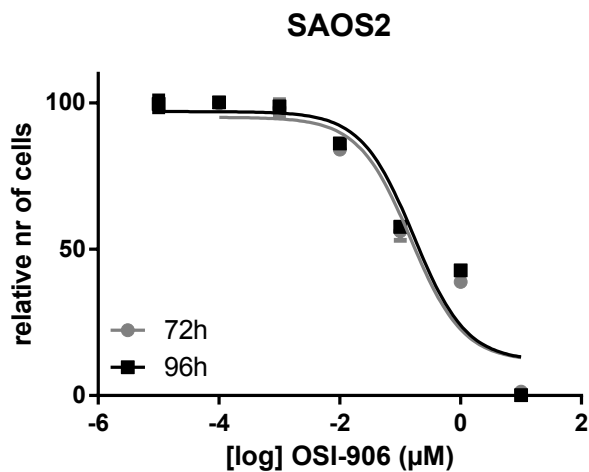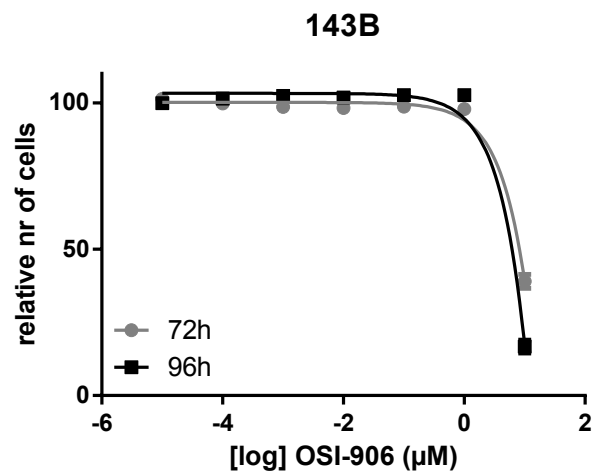

Supplement: Additional file 4 — Dose response curves up to toxic levels of OSI-906. Osteosarcoma cell lines were inhibited with different concentrations of OSI-906, for 72 (gray line) or 96 (black line) hours. [file 1471-2407-13-245-S4.pdf]
